# Strong Eukaryotic IRESs Have Weak Secondary Structure

**DOI:** 10.1371/journal.pone.0004136

**Published:** 2009-01-06

**Authors:** Xuhua Xia, Martin Holcik

**Affiliations:** 1 Department of Biology and Center for Advanced Research in Environmental Genomics, University of Ottawa, Ottawa, Canada; 2 Ottawa Institute of Systems Biology, University of Ottawa, Ottawa, Canada; 3 Apoptosis Research Center, Children's Hospital of Eastern Ontario, Ottawa, Canada; 4 Department of Pediatrics, University of Ottawa, Ottawa, Canada; 5 Department of Biochemistry, Microbiology and Immunology, University of Ottawa, Ottawa, Canada; Victor Chang Cardiac Research Institute, Australia

## Abstract

**Background:**

The objective of this work was to investigate the hypothesis that eukaryotic Internal Ribosome Entry Sites (IRES) lack secondary structure and to examine the generality of the hypothesis.

**Methodology/Principal Findings:**

IRESs of the yeast and the fruit fly are located in the 5′UTR immediately upstream of the initiation codon. The minimum folding energy (MFE) of 60 nt RNA segments immediately upstream of the initiation codons was calculated as a proxy of secondary structure stability. MFE of the reverse complements of these 60 nt segments was also calculated. The relationship between MFE and empirically determined IRES activity was investigated to test the hypothesis that strong IRES activity is associated with weak secondary structure. We show that IRES activity in the yeast and the fruit fly correlates strongly with the structural stability, with highest IRES activity found in RNA segments that exhibit the weakest secondary structure.

**Conclusions:**

We found that a subset of eukaryotic IRESs exhibits very low secondary structure in the 5′-UTR sequences immediately upstream of the initiation codon. The consistency in results between the yeast and the fruit fly suggests a possible shared mechanism of cap-independent translation initiation that relies on an unstructured RNA segment.

## Introduction

Translation initiation by Internal Ribosome Entry Site (IRES) RNA elements is an alternative mode of translation that is utilized by some viruses and a small subset of eukaryotic mRNAs. IRES-mediated translation initiation is believed to allow direct recruitment of the ribosome to the vicinity of the initiation codon, thus bypassing the requirement for m^7^G cap and its associated protein factors [1]–[3]. While IRES-mediated translation mechanism can explain how these viral and eukaryotic genes can be translated when cap-dependent translation machinery has been attenuated, the nature of IRESs and their molecular details are not fully understood. Although many cellular IRESs have been experimentally identified and an IRES database has been created, there has been no sequence similarity identified among the IRESs with the exception of mRNAs from closely related species [4]–[7]. The lack of observable sequence similarity has resulted in a widely held view that IRESs likely possess stable secondary structure allowing them to interact with the components of the translation machinery. While this is true for viral IRESs, this notion has never been critically evaluated for cellular IRESs. In fact, some of the published literature suggests that the lack of secondary structure may be important for cellular IRES activity [8]–[12]. For example, mutations in the IRES element of XIAP that changed the secondary structure of this IRES had no impact on the XIAP IRES activity [13]. Similarly, the activity of Apaf-1 IRES is dependent on the binding of two RNA binding proteins, PTB and unr, that change the structure of Apaf-1 IRES such that it permits ribosome landing (and consequent translation initiation) to a single stranded region [14]. Intriguingly, mutations that forced Apaf-1 IRES into an open configuration resulted in an increased IRES activity despite the inability of IRES to bind PTB and unr [14]. We therefore investigated whether IRES activity requires stable secondary structure in its RNA.

## Results

### Yeast IRESs exhibit weak secondary structure

**Figure 1:**
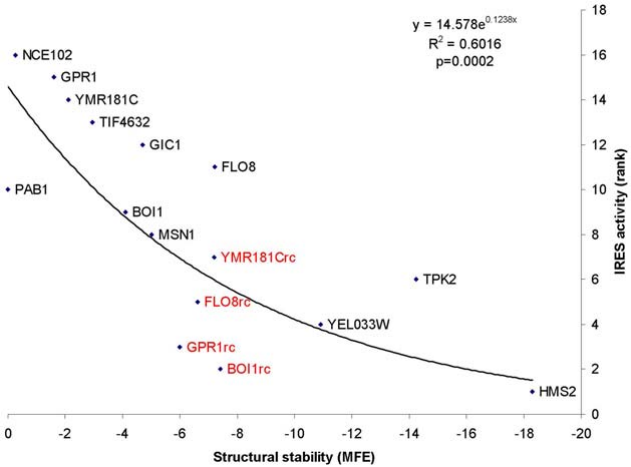
Negative correlation between IRES activity (measured as protein/mRNA of the reporter gene and ranked by the IRES strength from strongest to weakest [15]), and structural stability (measured as minimum free energy (MFE), kcal/mol) of yeast IRESs. The MFE is shown in reverse order because greater stability is associated with more negative MFE values. The reverse complements of four IRES-containing genes are colored in red. TIF4632 is the name in GenBank for gene *eIF4G2.*

We tested whether IRES activity depends on stable secondary structure by studying 12 yeast genes (*NCE102, GPR1, YMR181C, GIC1, FLO8, BOI1, MSN1, PAB1, eIF4G2, TPS2, HMS2,* and *YEL033W*) whose 5′UTRs differ dramatically in IRES activity [15]. The IRES activity in these genes was previously mapped to 60 nt immediately upstream of the initiation codon [15]. Also included in our analysis were the reverse complements of four of the experimentally identified yeast IRESs in *YMR181C, GPR1, FLO8,* and *BOI1* (designated as *YMR181Crc, GPR1rc, FLO8rc,* and *BOI1rc,* respectively). We found that the IRES activity of yeast IRESs is strongly associated with weak secondary structure measured by the minimum folding energy (MFE, in kcal/mol) of the 60 nt immediately upstream of the initiation AUG (Figure 1, r = −0.7756, p = 0.0002), contrary to the conventional belief that IRESs should have complex and stable secondary structure [4]–[7]. This result suggests that RNA segments with weak or no secondary structure immediately upstream of the initiation AUG can facilitate internal ribosome entry in yeast. This notion is further supported by the observation that the reverse complements of four of the identified IRESs have little IRES activity [15] and exhibit relatively stable secondary structure (Figure 1).

### A subset of Drosophila melanogaster IRESs also exhibits weak secondary structure

**Figure 2:**
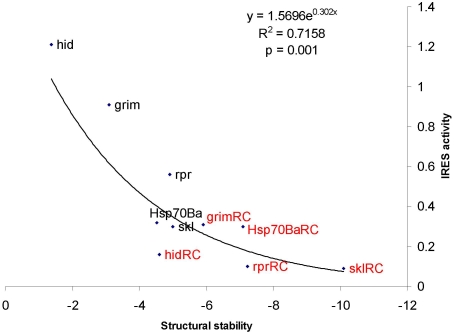
Negative correlation between relative IRES activity (Rluc/Fluc, derived from Figure 3b in [11]), and structural stability (measured as minimum free energy (MFE), kcal/mol) of *Drosophila* IRESs. The MFE is shown in reverse order because greater stability is associated with more negative MFE values. The reverse complements of the corresponding 60 nt 5′-UTRs are colored in red.

We wished to extend our observation that yeast IRES elements lack strong secondary structure to IRESs from other species. Previous publications [8], [11] investigated the IRES activity of the 5′-UTR sequences in five *Drosophila melanogaster* protein-coding genes, *rpr, hsp70, hid, grim* and *skl*, as well as the IRES activity of the reverse complement of the 5′-UTR sequences. We analyzed the 60 nt segment immediately upstream of the initiation codon of these genes and their reverse complements. We found that the strength of IRES activity of the fruit fly IRESs is also strongly associated with weak secondary structure (Figure 2, r = −0.8461, p = 0.001), consistent with the pattern observed with yeast data in Figure 1. The reverse complements of the 60 nt 5′-UTR sequences in the five fruit fly genes showed little or no IRES activity [8], [11] and all exhibited relatively stable secondary structure (Figure 2). The observation that the *skl* sequence with the least IRES activity also has the most stable secondary structure supports the general pattern (Figure 2). These results strengthen our conclusion that RNA segments with weak secondary structure immediately upstream of the initiation AUG can facilitate internal ribosome entry.

## Discussion

Past efforts in searching for structure conservation among cellular IRESs were unable to identify common feature(s) of cellular IRESs [6], [13]. The lack of sequence and structure conservation among reported IRESs has become one of the reasons for cellular IRESs to be criticized e.g. [16], [17]. Our finding that yeast and fruit fly IRESs are sequence segments devoid of strong secondary structure are similar to those suggested previously [9]–[12] and could explain why previous investigations did not identify common structural motifs in cellular IRESs. The two lines of evidence, from the unicellular yeast and the multicellular fruit fly, are quite consistent (Figures 1–2) and suggest a possible shared mechanism of cap-independent translation initiation. We wished to further extend this analysis to mammalian IRES elements described to date. However, given the diversity of experimental conditions and the reporter systems that were used to describe mammalian IRESs we were unable to compare many IRESs. When we performed structure-function analysis on 5 mammalian IRESs that were tested simultaneously [18] we did not find strong association between IRES activity and MFE. In particular, the 5′-UTR of XIAP exhibits the highest IRES activity among the five human genes examined, but its secondary structure is always the second weakest when secondary structure stability is measured by MFE with 60, 80, or 100 nt immediately upstream of the initiation AUG. This result is perhaps not surprising. The yeast IRESs were identified in response to the same stress (starvation induced differentiation) [15], and the *Drosophila* IRESs are found within a group of genes with similar function [8], [11]. It is therefore possible that these IRESs evolved under similar selective pressures and share a common feature such as weak secondary structure. In contrast, the human cellular IRESs tested by Nevins et al. [18] are quite distinct and the genes harbouring these elements are expressed under different cellular circumstances. Furthermore, the deletional and mutational analysis that has been performed on the mammalian IRES elements suggests that the mammalian IRESs are larger and more complex than the yeast or *Drosophila* IRESs. These key differences may explain the lack of correlation between the mammalian IRES activity and their secondary structure.

It is not clear how the structure-less IRESs would specifically engage ribosomes. Unlike the IRES of the intercistronic region of the cricket paralysis virus that is capable of directly interacting with the ribosome *via* its complex secondary structure [19], the other IRESs, both viral and eukaryotic, require various initiation factors and/or specific trans-acting factors, ITAFs, to facilitate the recruitment of the ribosome to the RNA [5]. Since the binding of virtually all ITAFs to RNA is quite promiscuous we may be unable to identify specific sequence elements common to IRESs, although it is possible that the IRES sequence segments devoid of secondary structure function through binding of specific ITAFs. We have made a similar observation previously when we identified novel IRES elements that share limited structural homology and ITAF binding sites with the IRES of XIAP [13].

## Materials and Methods

The annotated *Saccharomyces cerevisiae* genome, dated Sept. 17, 2007, was downloaded from ftp.ncbi.nih.gov/genomes/Saccharomyces_cerevisiae. The 60 nt immediately upstream of the initiation AUG was extracted using DAMBE [20], [21]. The 5′-UTRs are shown in Table S1 in supplementary online material. The annotated *Drosophila melanogaster* genome, dated May 14, 2008, was also downloaded from GenBank. For the five *D. melanogaster* whose 5′-UTR and the associated reverse complements have been studied for IRES activity [8], [11], we extracted the 60 nt immediately upstream of the initiation AUG and obtained their reverse complements. The sequences (60 nt immediately upstream of the initiation AUG and their reverse complements), are shown in Table S2 in supplementary online material.

The minimum folding energy (MFE) was computed using DAMBE which incorporates the function library of the Vienna RNA package [22], at 37°C, with no lonely pairs and with no GU pairs at the end of helices. The result was similar to that from the MFold server (http://www.bioinfo.rpi.edu/applications/hybrid/zipfold.php) [23], but the latter sometimes produced positive MFE values that are difficult to interpret. The relative rank of MFE remained the same when computed at higher or lower temperatures.

## Supporting Information

Table S1 (0.02 MB DOC)

Table S2 (0.02 MB DOC)
