# Supplementary material for: Strong Eukaryotic IRESs Have Weak Secondary Structure
Source: PLoS One. 2009 Jan 6;4(1):e4136. doi: 10.1371/journal.pone.0004136 (PMC2607549; doi:10.1371/journal.pone.0004136)
Supplement: Table S1 — (0.02 MB DOC) [file pone.0004136.s001.doc]

**Supplementary Table S1.** 60 nt immediately upstream of 12 yeast genes (Gilbert et al. 2007 [15]), together with the reverse complement (suffixed by “rc” in sequence name) of four of the yeast genes, in FASTA format.

>YEL033W 60 bases

CUCCUUCAAGGAAGCUGCUAGAACCGAUUAAACCGGUUAACAUCAUGGCAUGGGAUAUAA

>HMS2 60 bases

AUAUUGCCAGUCAGGGCAGUCGCCAAGUGUGUCUCUUUCGUGUAGCGACGCACGUCAGAC

>TPK2 60 bases

CAGCCGGCACAAACAGCAGCUUCACUCAGGUUAACUCACAUACUGUUGAAAAUUGUCGGU

>BOI1 60 bases

AUUUCAACAAAGUUCUAACUCGAGGUGACCGGAGGCCACUGUAAUAAUAAAAAAUAGAAG

>GPR1 60 bases

AAAAAAACGACAAACAAGUGAUCCGAAGUGUGACGAAUAAAGCAAACUCUCCAACUCAAA

>FLO8 60 bases

AAAAAUAAACACGAAGACGUUUAUAGACAUAAAUAAAGAGGAAACGCAUUCCGUGGUAGA

>PAB1 60 bases

UAUUCAAUAAAAACUCAAAAAAAAAAAUCCAAAAAAAACUAAAAAACCAAUAAAAAUAAA

>TIF4632 60 bases

UUUUACUGUUCCUGAAAGAAAGAAUUUAUUACAAAGAACAAUAGAUCAAUUGUAGGCACU

>GIC1 60 bases

UUUAUUGUUUAUAAUUCACUCUGAAUACUCGCUUAUUAAUUGUGUGAUAAAUUCUAAAGG

>YMR181C 60 bases

CCCUUAUAUUUACUCACCUUCCCCCUACUCUAAUUCUCCUGAUUUCAGAACAAAAAAAAA

>MSN1 60 bases

CUUGCUUAUAAGAAAAGAAACCAAAUCAGAAAAGGAGAUUAUUUCAAGGUAGGCAUCGAA

>NCE102 60 bases

AAAAAUCGGUUAAAAAAACUUUUCUUCUCAAAGCAUACCUAAUAACAAUAUAAUCCCAUA

>BOI1rc 60 bases

CUUCUAUUUUUUAUUAUUACAGUGGCCUCCGGUCACCUCGAGUUAGAACUUUGUUGAAAU

>GPR1rc 60 bases

UUUGAGUUGGAGAGUUUGCUUUAUUCGUCACACUUCGGAUCACUUGUUUGUCGUUUUUUU

>FLO8rc 60 bases

UCUACCACGGAAUGCGUUUCCUCUUUAUUUAUGUCUAUAAACGUCUUCGUGUUUAUUUUU

>YMR181Crc 60 bases

UUUUUUUUUGUUCUGAAAUCAGGAGAAUUAGAGUAGGGGGAAGGUGAGUAAAUAUAAGGG
