# Supplementary material for: Strong Eukaryotic IRESs Have Weak Secondary Structure
Source: PLoS One. 2009 Jan 6;4(1):e4136. doi: 10.1371/journal.pone.0004136 (PMC2607549; doi:10.1371/journal.pone.0004136)
Supplement: Table S2 — (0.02 MB DOC) [file pone.0004136.s002.doc]

**Supplementary Table S2.** 60 nt immediately upstream of the initiation AUG of the five *Drosophila melanogaster* genes [8,11], together with their reverse complement (suffixed by “RC” in sequence name), in FASTA format.

>Hsp70Ba 60 bases

UAUUGAAUACAAGAAGAGAAUACUUUCAACAAGUUACCGAGAAAGAAGAACUCACACACA

>grim 60 bases

AUUUUUUUAAAGAUUCAUACGUUUUGUAAAAUCACAUUUUGUAUUAAAUUAAAUACCGCC

>hid 60 bases

GGAAGGAAGGAAGCGGAUAAGGACAAAAAGGAAGCCAGCACACACACACACACCCACACA

>rpr 60 bases

GAAAGUUAUUGAGUCACUACCAGUUGUGUAAUUCCGAACGAGAAGAAAGAUAAACCAACA

>skl 60 bases

CUUGCCACACAUCGAAGUUAACAAGUGCCAUUUUUACGUAACAUUUUGAGCGACUCAAAU

>Hsp70BaRC 60 bases

UUCUCUUCUUGUAUUCAAUAAUUACUUCUUGGCAGAUUUCUGUAGUUGCAGUUGAUUUAC

>grimRC 60 bases

GUAUGAAUCUUUAAAAAAAUAUAUAACUUUAGUGUAGCAUAUAUUUUUAAUGUUUGAUUU

>hidRC 60 bases

UUAUCCGCUUCCUUCCUUCCUGCACUUUGUUGGCACUUUGCUCUUCUUGUGAUUGUUCUU

>rprRC 60 bases

GUAGUGACUCAAUAACUUUCUCUUCUGCACAAUGAAUAUUUAUCGAGUUAGUUCUUUGUU

>sklRC 60 bases

UAACUUCGAUGUGUGGCAAGGUAUUUCCUGAUUUUCCAGAUCUGAACUCUUGGCGAGUUG
